# Letter in reply: Linear IgA bullous dermatosis treated with dupilumab in a pediatric patient with glucose-6-phosphate dehydrogenase deficiency

**DOI:** 10.1016/j.jdcr.2023.11.025

**Published:** 2023-12-07

**Authors:** Sonal Muzumdar, Lorin A. Bibb, Brett Sloan, Michael Murphy, Mary Wu Chang

**Affiliations:** a Department of Dermatology, University of Connecticut Health Center, Farmington, Connecticut; b Department of Dermatology, Mayo Clinic, Rochester, Minnesota; c Department of Pediatrics, University of Connecticut Health Center, Farmington, Connecticut

**Keywords:** chronic bullous dermatosis of childhood, dupilumab, linear IgA bullous dermatosis, pediatric dermatology

*To the Editor:* In the recently published article “Successful treatment of linear immunoglobulin A bullous dermatosis with dupilumab in a pediatric patient,” Almuhanna et al^1^ described the first reported case to date of linear IgA bullous dermatosis (LABD) successfully treated with dupilumab in a 7-year-old patient.

We would like to report the second case of childhood LABD successfully treated with dupilumab. A 14-month-old male child with a history of atopic dermatitis presented with a 2-week history of vesicles that started on the bilateral cheeks. He had been treated with topical mupirocin and oral cephalexin for presumed impetigo without improvement. At presentation, there were scattered erythematous, eroded plaques, and tense bullae in a “crown of jewels” arrangement on the cheeks, trunk, and bilateral upper and lower extremities (Fig 1, *A, B*). The lesions were pruritic. The patient was afebrile and otherwise well-appearing. A lesional punch biopsy was obtained from the patient’s thigh for hematoxylin-eosin (H&E) staining and a perilesional biopsy was taken for direct immunofluorescence (DIF). H&E stain revealed epidermal degeneration with a mixed inflammatory infiltrate including neutrophils and eosinophils. DIF demonstrated linear, homogenous IgA deposition along the dermoepidermal junction without IgG or C3, consistent with chronic bullous dermatosis of childhood (Fig 2). Laboratory testing revealed a significantly reduced glucose-6-phosphate dehydrogenase (G6PD) level of 1.8 U/g Hb (reference: 9.9-16.6 U/g Hb). Because dapsone was contraindicated owing to G6PD deficiency, he started receiving prednisolone 1 mg/kg/d and dupilumab 200 mg every 4 weeks. Three months after starting dupilumab, the patient was successfully tapered off prednisolone. He has continued receiving dupilumab monotherapy for 3 months with complete clearance of disease.

**Fig 1:**
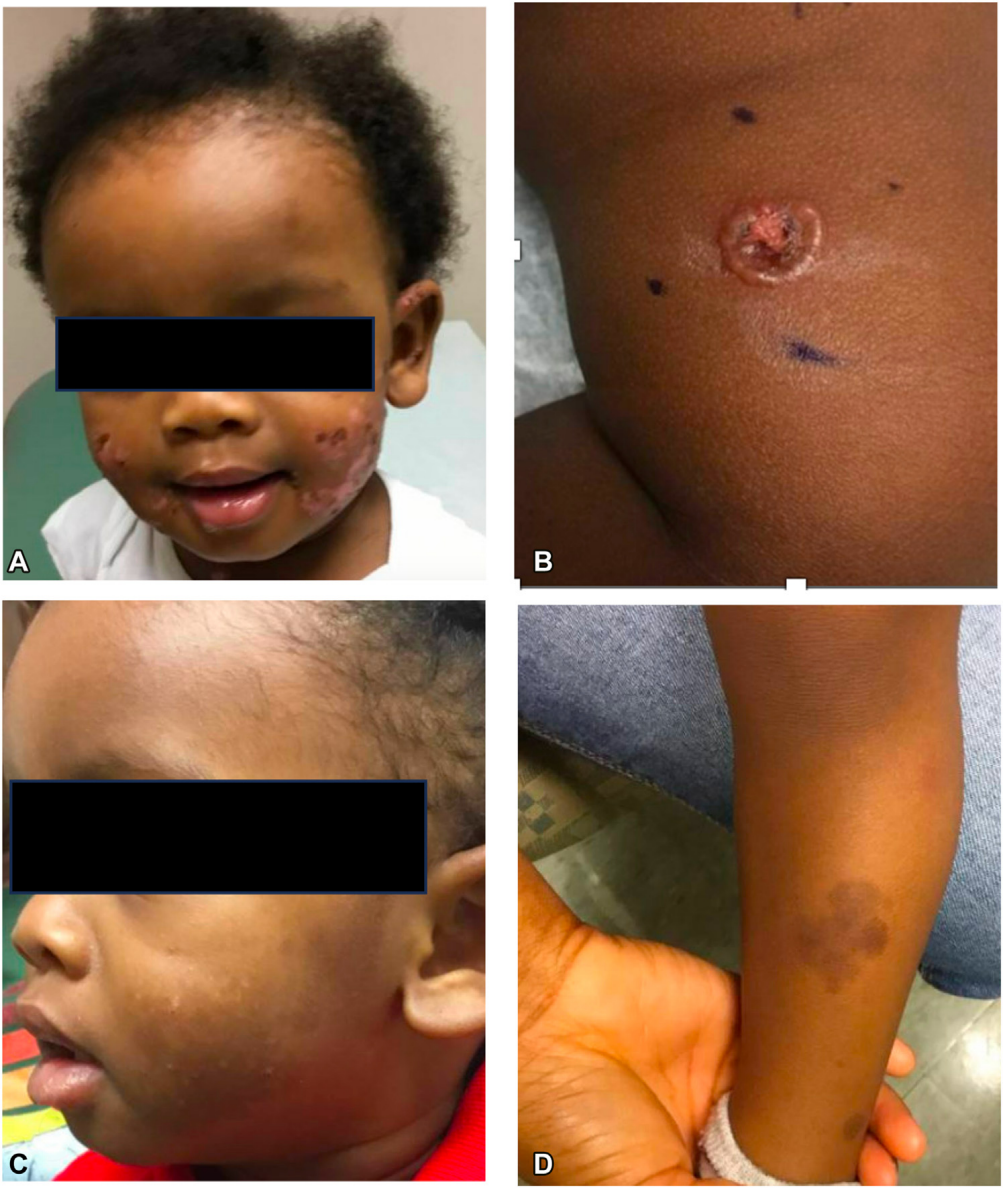
Before dupilumab therapy, the patient had **(A)** vesicles and eroded papules on the bilateral cheeks and **(B)** bullae in a “crown of jewels” distribution on the legs. After 6 months of therapy with dupilumab, there was clearing of lesions on **(C)** the cheeks and **(D)** lower extremities, with residual postinflammatory hyperpigmentation.

**Fig 2:**
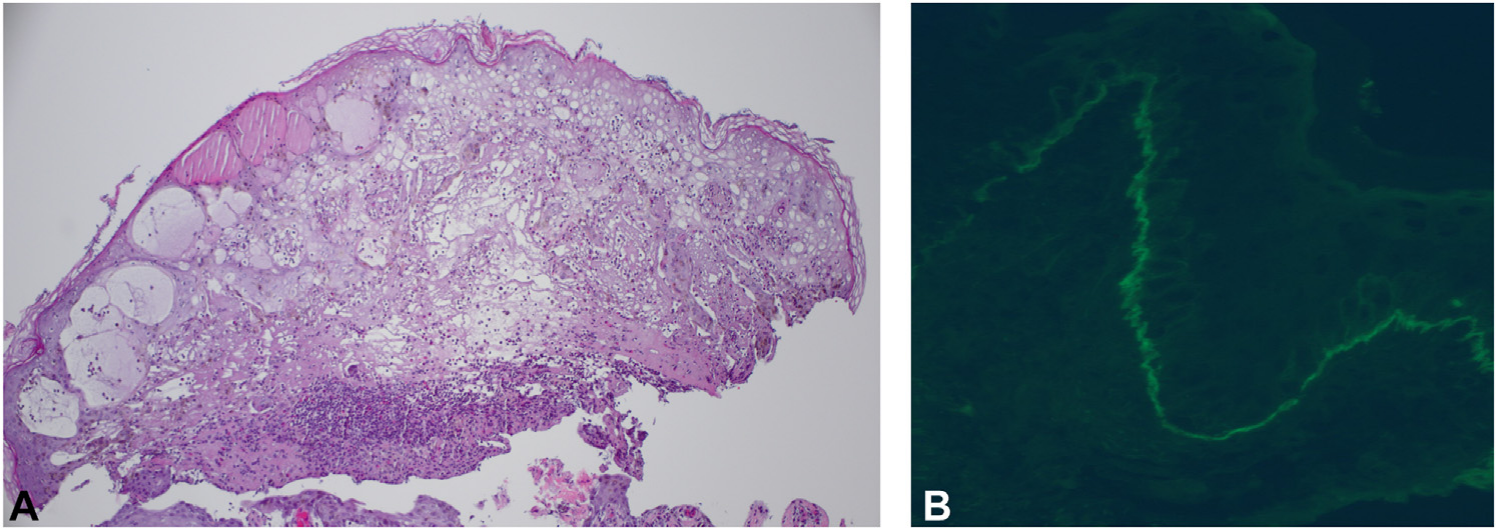
**A,** Hematoxylin-eosin–stained biopsy of the lower portion of the leg demonstrated epidermal degenerative changes with a mixed inflammatory infiltrate of neutrophils and eosinophils. **B,** Direct immunofluorescence revealed linear, homogenous IgA deposition along the dermoepidermal junction.

LABD is a rare bullous dermatosis with an estimated incidence of 0.2 to 2.3 cases per million annually.^2^ Dapsone is the first-line treatment and may be used in conjunction with oral corticosteroids.^2^ However, dapsone may induce hemolytic anemia and is thus avoided in patients with risk factors, including G6PD deficiency. In the case presented by Almuhanna et al,^1^ the patient had anemia, which precluded the use of dapsone. Similarly, our patient had a low G6PD level, which put him at a high risk of hemolytic anemia. In both cases, dupilumab was started in conjunction with a systemic corticosteroid taper. Corticosteroids were tapered off, and dupilumab monotherapy resulted in excellent disease control. Dupilumab is a monoclonal antibody that binds interleukin 4 receptor alpha, leading to the inhibition of interleukins 4 and 13.^3^ The mechanism by which dupilumab works in the treatment of LABD is unknown, but it may be due to the downregulation of the type 2 helper T-cell pathway and associated inflammatory cytokines. Further study is warranted to fully understand the role of dupilumab in the treatment of LABD, but it shows promise as an appropriate treatment option in patients for whom dapsone may not be tolerated.

## Conflicts of interest

None disclosed.
